# Subtrochanteric fracture after core decompression for osteonecrosis of the femoral head: a case report and literature review

**DOI:** 10.1186/s12891-024-07536-5

**Published:** 2024-05-29

**Authors:** Shihong Li, Chen Huang, Wei Wang, Song Chen, Bing Deng, Li Yin, Yida Amu, Lijuan Ye, Dan Jing, Benjing Song, Qingyun Xie, Dongfa Liao

**Affiliations:** The General Hospital of Western Theater Command, Chengdu, China

**Keywords:** Osteonecrosis of the femoral head, Core decompression, Subtrochanteric fracture

## Abstract

**Background:**

Osteonecrosis of the femoral head (ONFH) is a common clinical disease. Improper treatment can lead to femoral head collapse and hip joint dysfunction. Core decompression is particularly important for early ONFH. However, subtrochanteric fractures after core decompression cause some clinical problems.

**Case presentation:**

This article describes a 34-year-old male patient with early ONFH. After core decompression, he suffered a subtrochanteric fracture of the femur while bearing weight on the affected limb when going up stairs. He was subsequently treated with open reduction and intramedullary nail fixation.

**Conclusion:**

When core decompression is used to treat ONFH, the location or size of the drill hole, whether a tantalum rod or bone is inserted, and partial weight-bearing of the affected limb may directly affect whether a fracture occurs after surgery. It is hoped that this case report can provide a reference for clinical orthopedic surgeons in the treatment of early ONFH.

## Background

Osteonecrosis of the femoral head (ONFH) is a common clinical disease. Fukushima et al. reported different causes of non-traumatic ONFH in Japan, of which 51% were steroid-induced, 31% were alcohol-induced, and 15% were idiopathic, the use of steroids is a key factor in the pathogenesis of non-traumatic ONFH [1]. Kang et al. reported that the causes of ONFH in South Korea were steroid-induced in 14.6%, alcohol-induced in 32.4%, idiopathic in 51.4%, and traumatic in 1.6% [2]. However, among Chinese ONFH patients, the study found that 24.1% of patients were steroid-induced, 30.7% were alcohol-induced, 16.4% were trauma-induced, and 28.8% were idiopathic [3] At the same time, domestic scholars collected a large number of ONFH cases and found that steroid-induced ONFH accounted for 26.84%, alcoholics accounted for 37.15%, and traumatic ONFH accounted for 15.73% [4]. Although a large number of scholars have done epidemiological research on ONFH, regional differences, ethnic differences, customs and cultural differences will all affect the pathogenesis, which still requires more research to deepen the understanding of ONFH.

The current staging system for ONFH is revised based on the 1994 Association Research Circulation Osseous (ARCO) classification [5]. Stage I: Normal X-rays, but positive MRI or bone scan. Stage II: X-ray abnormalities (focal osteoporosis, osteosclerosis, or cystic changes of the femoral head), but no subchondral fractures, necrotic fractures, or flattening of the femoral head. Stage III: Fracture of subchondral or necrotic area, stage IIIA (femoral head depression ≤ 2 mm), stage IIIB (femoral head depression > 2 mm). Stage IV: Radiographic evidence of osteoarthritis with joint space narrowing, acetabular changes, and/or joint destruction.

Improper treatment can lead to femoral head collapse and hip joint dysfunction, so hip-preserving treatment for early ONFH is very important [6]. Studies have found that alendronate, a bisphosphonate drug, appears to prevent early collapse of the femoral head [7]. Core decompression therapy alone or core decompression combined with autologous bone therapy can have good therapeutic effects on femoral head necrosis [8]. Platelet-rich plasma (PRP) can be able to induce angiogenesis and osteogenesis to accelerate bone healing and inhibit the inflammatory response of necrotic lesions to treat ONFH [9]. Free vascularized fibular transplantation can provide support for the articular surface, reduce intraosseous pressure, remove and replace necrotic tissue, and improve the biological microenvironment of the area. This is another important hip-preserving method for the treatment of femoral head necrosis [10]. These treatments include medical therapy, core decompression, bone grafting, platelet-rich plasma and free fibular grafting [7–10], where core decompression can both increase blood flow in the diseased area and reduce intraosseous pressure, thereby reducing pain [11, 12], so it is a common clinical method to treat early ONFH. However, this method also has some complications. This article introduces a patient who suffered a subtrochanteric fracture after core decompression. We hope it can be used as a reference for clinical orthopedic surgeons.

## Case report

**Fig. 1:**
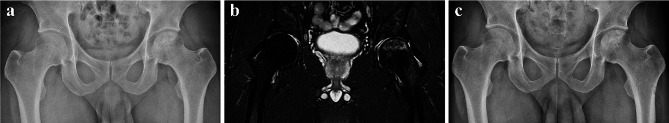
**a** shows the patient’s preoperative pelvic plain radiograph, **b** shows the preoperative hip MRI, and **c** shows the postoperative pelvic plain radiograph

**Fig. 2:**
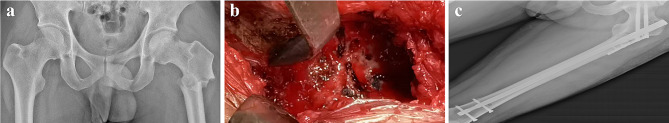
**a** shows the patient’s preoperative plain radiograph of the pelvis, **b** shows the intraoperative situation, and **c** shows the postoperative full-length plain radiograph of the femur

The patient went to the hospital with “left hip pain for 4 months” and was treated with non-steroidal analgesics outside the hospital for 4 months. The symptoms did not improve after conservative treatment. The patient is a 34-year-old male, BMI 23.5 kg/m². The patient had no history of oral steroid drugs or alcoholism. He only drank beer occasionally, 250 ml-500 ml/time. The patient’s femoral head necrosis was most likely to be idiopathic. Imaging showed avascular necrosis of the left femoral head (ACRO stage II). He underwent percutaneous drilling decompression of the left femoral head under spinal anesthesia, the perioperative imaging is shown in Fig. 1. During core decompression, we performed core decompression with the help of Mobile C-arm X-ray unit, we used 2.5 mm diameter K-wire for drilling and made a total of 4 canals. Postoperative analgesia was provided, partial weight-bearing walking with the help of crutches, and regular follow-up was performed. One month after the operation, the patient’s left lower limb was completely weight-bearing when going up the stairs, and then there was severe pain in the left thigh. Imaging showed a subtrochanteric fracture of the left femur. Open reduction and intramedullary nailing and internal fixation were performed under general anesthesia. Perioperative imaging is shown in Fig. 2.

## Discussion and conclusions

Insufficient blood supply to the femoral head will lead to tissue death and subsequent collapse of the bone under the load-bearing cartilage [13]. After the collapse of the femoral head, hip replacement is the last treatment option, so intervention for early ONFH is particularly important. Core decompression is a minimally invasive surgical technique that requires an access hole in the lateral cortex of the femur, which may predispose patients to subtrochanteric fractures. According to a clinical meta-analysis, the risk of subtrochanteric fracture is approximately 0.9% [8], and studies have found that a duct diameter of 8 mm has been shown to weaken the bone compared with smaller duct sizes [14]. The location of the tube is also important, with more distal origins leading to a higher risk of subtrochanteric fractures [15]. Studies have found that the optimal entry point for core decompression of the femur is in the proximal subtrochanteric area to reduce the risk of subtrochanteric fracture. When the starting position is located at the more distal femur, the risk of femoral fracture increases in turn [16].

Currently, treatments such as platelet-rich plasma infusion [17], stem cell transplantation [18], porous tantalum rod implantation [19], and quadratus femoris pedicle bone transplantation [20] are often used together with core decompression to treat early ONFH, they can enhance tissue repair and mechanical strength in necrotic areas, prevent further collapse of the femoral head, and improve hip joint pain and function. Studies have found that synthetic bone grafts fill core decompression tunnels, which can reduce the occurrence of subtrochanteric fractures [21]. The advantages of bone grafting may be related to its good tissue healing and bone remodeling [11]. Tantalum rod implantation can also provide structural support for the femoral head and subtrochanter after core decompression. When the tantalum rod is removed, fracture may occur under the femoral trochanter [22].

In summary, the occurrence of subtrochanteric fracture after core decompression may be related to the location and size of the hole and whether the graft is inserted. Perhaps during the operation, the drilling position was close to the femoral trochanter, and the patient was instructed to partial weight bearing after the operation, so fracture may not occur after surgery. At the same time, the insertion of tantalum rods or bone after core decompression may also be a way to reduce fracture. It is hoped that this case report can provide a reference for clinical orthopedic surgeons in the treatment of early ONFH.

## Data Availability

The data used and/or analyzed during the current study are available from the corresponding author on reasonable request.

## Abbreviations

ACRO: Association Research Circulation Osseous
ONFH: Osteonecrosis of the femoral head
PRP: Platelet-rich plasma
